# Three-Dimensional Ordered Porous SnO_2_ Nanostructures Derived from Polystyrene Sphere Templates for Ethyl Methyl Carbonate Detection in Battery Safety Applications

**DOI:** 10.3390/nano15151150

**Published:** 2025-07-25

**Authors:** Peijiang Cao, Linlong Qu, Fang Jia, Yuxiang Zeng, Deliang Zhu, Chunfeng Wang, Shun Han, Ming Fang, Xinke Liu, Wenjun Liu, Sachin T. Navale

**Affiliations:** 1 College of Materials Science and Engineering, Guangdong Provincial Key Laboratory of New Energy Materials Service Safety and Guangdong Research Center for Interfacial Engineering of Functional Materials, Shenzhen Key Laboratory of Special Functional Materials, Shenzhen University, Shenzhen 518055, China; pjcao@szu.edu.cn (P.C.); qulinlong2022@email.szu.edu.cn (L.Q.); chiafang@szu.edu.cn (F.J.); zengyx@szu.edu.cn (Y.Z.); cfwang@szu.edu.cn (C.W.); hanshun@szu.edu.cn (S.H.); m.fang@szu.edu.cn (M.F.); xkliu@szu.edu.cn (X.L.); liuwj@szu.edu.cn (W.L.); 2 Nanomaterials and Sustainable Chemical Technologies (NanoTech), Department of Inorganic Chemistry, Faculty of Sciences, University of Granada, 18071 Granada, Spain

**Keywords:** battery safety, electrolyte leakage, ordered porous SnO_2_ nanostructures, gas sensor, EMC detection

## Abstract

As lithium-ion batteries (LIBs) gain widespread use, detecting electrolyte–vapor emissions during early thermal runaway (TR) remains critical to ensuring battery safety; yet, it remains understudied. Gas sensors integrating oxide nanostructures offer a promising solution as they possess high sensitivity and fast response, enabling rapid detection of various gas-phase indicators of battery failure. Utilizing this approach, 3D ordered tin oxide (SnO_2_) nanostructures were synthesized using polystyrene sphere (PS) templates of varied diameters (200–1500 nm) and precursor concentrations (0.2–0.6 mol/L) to detect key electrolyte–vapors, especially ethyl methyl carbonate (EMC), released in the early stages of TR. The 3D ordered SnO_2_ nanostructures with ring- and nanonet-like morphologies, formed after PS template removal, were characterized, and the effects of template size and precursor concentration on their structure and sensing performance were investigated. Among various nanostructures of SnO_2_, nanonets achieved by a 1000 nm PS template and 0.4 mol/L precursor showed higher mesoporosity (~28 nm) and optimal EMC detection. At 210 °C, it detected 10 ppm EMC with a response of ~7.95 and response/recovery times of 14/17 s, achieving a 500 ppb detection limit alongside excellent reproducibility/stability. This study demonstrates that precise structural control of SnO_2_ nanostructures using templates enables sensitive EMC detection, providing an effective sensor-based strategy to enhance LIB safety.

## 1. Introduction

Over the past decade, lithium-ion batteries (LIBs) have emerged as one of the highly preferred energy storage technologies in various applications, ranging from electric vehicles and portable electronics to renewable energy devices [1,2]. In particular, LIBs’ high energy density, compact system, and extended cycle life made them ideal for current power demands [1]. However, as the use of LIBs continues to grow, their safety concerns are also increasing. For example, the risk of thermal runaway (TR) is regarded as one of the most critical safety issues of LIBs [3]. Typically, a TR in LIBs arises when the battery overheats uncontrollably because of excessive heat that can lead to fire and explosion [3,4,5]. Moreover, it is usually triggered by overcharging, an internal short circuit, and internal degradation processes, during which various gaseous byproducts—typically volatile organic compounds (VOCs)—are released due to the breakdown of battery components, particularly the electrolyte [1,2,3]. In LIBs, the electrolyte is key and is generally composed of one or more lithium salts dissolved in a mixture of organic carbonate solvents, such as ethyl methyl carbonate (EMC) [4,5]. The low viscosity and high ionic conductivity of EMC make it a widely used solvent for commercial LIBs electrolytes, leading to dynamic lithium-ion transport [5,6]. Notably, under early thermal stress conditions in LIBs, EMC can begin to evaporate prior to the onset of TR process, making it an effective indicator of early-stage electrolyte instability. Therefore, a targeted early warning signal for LIBs safety could be achieved by detecting EMC vapor. In this regard, in order to enhance the safety and reliability of LIBs, there is an increasing emphasis on integrating gas sensing technologies that can detect early signs of failure and prevent potential accidents [4,5,6]. However, despite its importance, EMC detection has not been fully explored in the context of gas sensing. To date, the detection of carbonate is reported using traditional gas chromatography–mass spectrometry and nuclear magnetic resonance spectrometers [6,7]. Although these detection techniques provide high accuracy, they are not suitable for real-time embedded monitoring within battery systems because of their bulkiness, high cost, and incompatibility. In this respect, chemiresistive gas sensors offer a more practical and efficient alternative to aforesaid traditional gas detection methods due to their compact size, low cost, and simple design, allowing timely detection and improved battery management [4,6,8]. In addition, their resistance-based gas detection allows for real-time monitoring of VOCs, making them compatible for battery system integration. Until now, chemiresistive sensors based on semiconducting metal oxides (SMOs) are widely used for detecting VOCs and toxic gases in practical applications [9]. Among various SMOs, tin oxide (SnO_2_)—a typical n-type semiconductor—has gained significant attention due to its high optical transparency (bandgap of ~3.5–3.8 eV) and tunable properties, making it a key material in applications such as perovskite solar cells, transparent conductors, UV photodetectors, and gas sensors [10,11,12]. Among its various applications, particular attention has been given to gas sensing, where it is regarded as a promising sensing material due to its low cost, high sensitivity, excellent thermal stability, and tunable surface/electronic properties [12]. Moreover, the sensing performance of SnO_2_-based sensors can be significantly improved by tailoring key material properties, such as surface area, porosity, crystal size, and oxygen vacancies [6,12]. In particular, controlling the nanostructure of SnO_2_ leads to increased active surface area, which promotes efficient gas adsorption and charge transfer. These tunable features of SnO_2_ make it a very sensitive and adaptable material, which is ideal for early detection of electrolyte vapors in LIB safety applications. It is important to note that although SnO_2_-based materials have been widely used for detecting various VOCs and toxic gases, their usage in detecting carbonate-based vapors remains understudied. Specifically, there are very few reports in the literature on EMC sensing using sensors based on SMOs. For example, Su et al. developed a SnO_2_ nanobox-based gas sensor, which showed a response of 32.46 to 20 ppm EMC at 140 °C [6]. The same research group also reported the detection of EMC using octahedral hollow SnO_2_ nanocages at 140 °C [13]. In another study, Liu et al. explored the EMC sensing properties of Pd-doped WO_3_ and reported a response of 17.8 to 10 ppm EMC at 275 °C [14]. Despite these efforts, research on SMOs-based sensors for EMC detection remains limited and requires further exploration. Therefore, given the importance of carbonate-based vapors as early indicators of TR in LIBs, our study focuses on utilizing SnO_2_-based nanostructures for the detection of electrolyte-based carbonate vapors, in particular EMC, aiming to advance battery safety monitoring technologies.

In this study, ordered porous SnO_2_ nanostructures were synthesized by means of a self-assembly method using three-dimensional polystyrene spheres (3D PS) of varying diameters as templates and different precursor concentrations [15]. Templating techniques are well known for fabricating nanostructured materials with controlled morphology, porosity, and surface area. Among them, 3D PS nanoparticle templates enable simple synthesis of highly porous SMO nanostructures via self-assembly, offering high surface area and tunable properties ideal for gas sensing [6,15]. Therefore, to examine the effect of SnO_2_ nanostructures on sensor performance, we synthesized them using PS templates of varying sizes. The crystallographic phase and morphological features of the resulting 3D ordered porous SnO_2_ nanostructures were systematically characterized and described. The effects of varying PS template sizes and different precursor concentrations on the sensor performance were systematically studied and compared with those of a template-free sample. In particular, comprehensive gas sensing measurements were carried out to evaluate the performance of the SnO_2_ sensors in detecting VOCs, particularly EMC, emitted during the early TR process of LIBs. Herein, compared with other carbonates, EMC was selected for sensor studies due to its higher flash point, lower toxicity, and chemical stability, making it a safer and more practical solvent [6]. In addition, given its widespread use in LIB electrolytes for thermal compatibility with Li salts and additives, EMC also serves as an appropriate target vapor for early detection of electrolyte leakage and TR in sensor-based systems [5,6].

## 2. Materials and Methods

*Chemicals:* All the reagents/chemicals, such as tin (IV) chloride pentahydrate (SnCl_4_·5H_2_O), isopropanol (C_3_H_8_O), hydrogen peroxide (H_2_O_2_, 30%), sulfuric acid (H_2_SO_4_), ethanol (C_2_H_5_OH), and acetone (C_3_H_6_O), used during synthesis procedure were of pure analytical grade and used as received. PSs of different sizes were acquired from Beijing Zhongke Keyou company, Beijing, China. Deionized (DI) water was used during the synthesis of materials.

*Pretreatment of silicon (Si) substrates*: Initially, the monocrystalline Si wafers (1 cm × 1 cm) were ultrasonically cleaned in C_3_H_6_O and anhydrous C_2_H_5_OH for 10 min each to eliminate organic and particulate contaminants. Following cleaning, in order to improve surface hydrophilicity and wettability, the Si wafers were immersed in piranha solution (a mixture of concentrated H_2_SO_4_ and H_2_O_2_ at a volume ratio of 7:3) for 1 hr. Afterwards, the wafers were cleaned yet again thoroughly with DI water and dried in an oven at 60 °C for 3 h. The dried Si wafers were then stored for further use.

*Self-assembly of 3D ordered PS templates*: To fabricate the PS templates, 100 μL of 5 wt% PS suspension with different particle sizes (200 nm, 500 nm, 700 nm, 1000 nm, and 1500 nm) was mixed with 100 μL of C_3_H_8_O and ultrasonicated for 20 min to ensure uniform dispersion. The resultant suspension was then drop-casted vertically onto the pretreated Si wafers to ensure the uniform distribution of droplets. Subsequently, these Si wafers were dried in an oven at 50 °C, resulting in the formation of ordered PS templates.

**Scheme 1:**
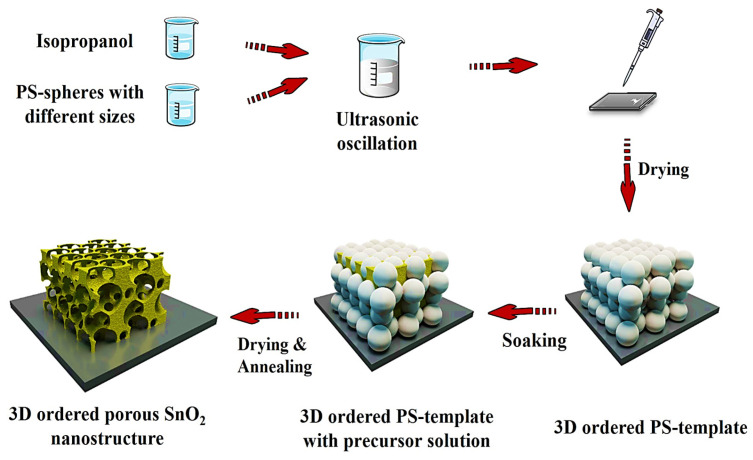
Synthesis scheme of 3D ordered porous SnO_2_ nanostructures with PS templates (precursor: SnCl_4_·5H_2_O).

*Growth of ordered porous SnO_2_ nanostructures*: A series of five precursor solutions with concentrations of 0.2, 0.3, 0.4, 0.5, and 0.6 mol/L were prepared by dissolving appropriate amounts of SnCl_4_·5H_2_O in 16 mL of anhydrous C_2_H_5_OH. These mixtures were subjected to magnetic stirring at room temperature for 30 min to ensure complete dissolution of the precursor material. In the beginning, in order to study the effect of template particle size on gas sensing performance, 50 μL of as-prepared and optimized 0.4 mol/L SnCl_4_·5H_2_O solution was drop-casted onto the PS templates with varying particle sizes of 200, 500, 700, 1000, and 1500 nm. These samples were then dried at 60 °C for 12 h, followed by annealing in a tube furnace at 550 °C for 2 h (with a heating rate of 2 °C/min) to eliminate the PS templates and transform them into ordered SnO_2_-X1 materials (here, X1 denotes the PS particle size). Afterwards, to examine the effect of precursor concentration, 50 μL SnCl_4_·5H_2_O solutions with different concentrations (0.2 to 0.6 mol/L) were drop-casted onto PS templates with a 1000 nm sphere size. These were also dried and annealed under the same conditions to produce SnO_2_-X2 samples (here, X2 represents SnCl_4_·5H_2_O concentration). For comparison, 50 μL of optimized 0.4 mol/L SnCl_4_·5H_2_O solution was drop-casted directly onto a blank pretreated Si wafer (without a PS template), then dried and annealed under the same conditions to obtain the SnO_2_ template-free sample (labelled as SnO_2_-TF). The SnO_2_ samples, prepared using PS templates of various sizes (200–1500 nm), are termed as SnO_2_-200, SnO_2_-500, SnO_2_-700, SnO_2_-1000, and SnO_2_-1500, corresponding to the respective PS template size. Similarly, the SnO_2_ samples, labelled SnO_2_-0.2 to SnO_2_-0.6, were synthesized using precursor concentrations ranging from 0.2 to 0.6 mol/L. The schematic presentation of the synthesis of ordered SnO_2_ nanostructures is shown in Scheme 1.

*Characterization techniques*: The crystal structure of the samples was examined by a Bruker D8 Advance X-Ray diffractometer (Bruker, Billerica, MA, USA) with CuK_α_ radiation (λ = 0.15406 nm, 2θ range of 20° to 80°). Surface morphology features of the samples were studied with a Hitachi SU-70 (Hitachi, Tokyo, Japan) field emission scanning electron microscope (FE-SEM). Elemental composition was analyzed using an energy-dispersive X-Ray spectroscopy (EDS) detector combined with the SU-70 FE-SEM (Hitachi High-Tech Corporation, Tokyo, Japan). High-resolution imaging of the sample were carried out using an FEI Titan Themis transmission electron microscope (TEM) (FEI, Hillsboro, OR, USA). The surface composition and oxidation states of the samples were investigated using a Thermo Scientific K-Alpha X-Ray photoelectron spectrometer (XPS) (Thermo Fisher Scientific, Waltham, MA, USA). The specific surface area and porosity of the samples were measured by a Micromeritics ASAP 2020 surface area (Micromeritics Instrument Corporation, Norcross, GA, USA) and porosity analyzer based on the BET (Brunauer–Emmett–Teller) method. The BJH method (Barrett–Joyner–Halenda) was used to determine the pore size distribution.

*Sensor fabrication and measurement*: The gas sensing tests on the fabricated SnO_2_ samples were conducted using the CGS-8 integrated gas sensing system (Beijing Elite Technology Co. Ltd., Beijing, China), as detailed in our earlier reports, which supports real-time measurement of up to eight sensors in a detection range of 1 Ω to 4 GΩ [16,17,18]. Temperature control and measurement sections are part of the system to ensure accurate testing. The sensor substrate consists of a ceramic tube that has a Ni-Cr wire inside to control the temperature of tube. The ceramic tube has Au electrodes at each end, connected via Pt wires to ensure electrical contacts. Our current sensing system is not compatible with direct measurements of film-type samples. Therefore, to enable evaluation of the sensing measurements, the SnO_2_ powder samples were carefully scraped from the Si substrates and used as sensing materials. In particular, for sensor fabrication, an appropriate amount of SnO_2_ powder samples were ground in a mortar to form a slurry/paste, after which its uniformity was adjusted with DI water or C_2_H_5_OH. This slurry/paste was later coated onto the surface of the ceramic tube by a fine pointed brush and dried in air. Finally, prior to sensing studies, the sensor is aged on a bench (Beijing Elite AS-20) for 24 h to stabilize both the material adhesion and the electrical contacts, assuring resistance stabilization. Herein, a data acquisition system was used to record the change in electrical resistance of the sensors when they were exposed to different gases. Sensor response (R) was calculated as: R = R_a_/R_g_ (for reducing gases) and R = R_g_/R_a_ (for oxidizing gases), where R_a_ and R_g_ are the sensor resistance values in the presence of the air and the target gas, respectively. Response and recovery times of the sensor were calculated using the previous reports [16,17,18]. Response time is defined as the time taken to reach 90% of the total resistance change upon gas exposure, while recovery time is the time taken to return to 90% of the baseline after the gas removal.

## 3. Results and Discussion

### 3.1. XRD Analysis

**Figure 1:**
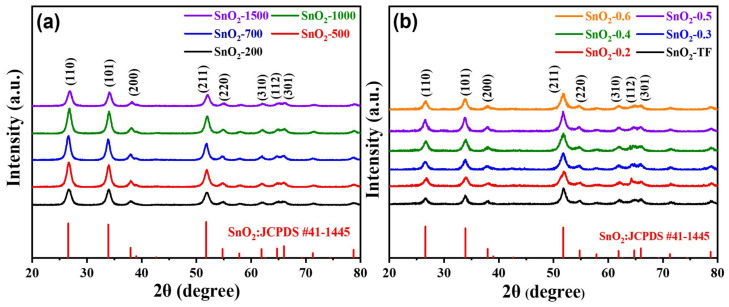
XRD patterns of (**a**) SnO_2_-X1 samples obtained from different 3D PS template sizes ranging 200–1500 nm (with 0.4 mol/L SnCl_4_·5H_2_O) and (**b**) SnO_2_-X2 samples obtained from different precursor concentrations ranging 0.2–0.6 mol/L (with a 1000 nm PS sphere size).

The XRD patterns of the SnO_2_-X1 and SnO_2_-X2 samples prepared using 3D PS templates of different sizes (SnO_2_-200 to SnO_2_-1500) and varying precursor concentrations (SnO_2_-0.2 to SnO_2_-0.6) are shown in Figure 1a and Figure 1b, respectively. As depicted in Figure 1, all XRD patterns appear as nearly matching, exhibiting characteristic diffraction peaks of the crystalline tetragonal phase of SnO_2_ (JCPDS No. 41-1445), indicating the formation of the same phase across all samples without any structural differences [6,12].

The XRD patterns of SnO_2_-X1 and SnO_2_-X2 samples exhibit prominent diffraction peaks located at 2θ values of 26.64°, 33.85°, 37.96°, 51.90°, 54.88°, 61.70°, 64.75°, and 66.98°, which are indexed to the (110), (101), (200), (211), (220), (310), (112), and (301) crystal planes of SnO_2_, respectively. Notably, all the samples showed high phase purity, as there were no additional peaks that corresponded to impurities detected, indicating that the materials synthesized both with and without PS templates (SnO_2_-TF) yielded pure-phase SnO_2_. Moreover, although all samples showed the characteristic diffraction peaks of SnO_2_, noticeable broadening of these peaks was evident. Such a peak broadening is generally known to be caused by a decrease in crystallite size and may also suggest a higher density of structural defects within the lattice [19]. From a gas sensing viewpoint, smaller crystallite sizes and higher surface defect densities are typically beneficial, as they boost the surface reactivity of the sensing material [19,20]. Therefore, these structural characteristics are expected to be advantageous in the gas sensing performance of the SnO_2_ nanostructures.

### 3.2. Surface Morphology, Elemental, and Surface Area Analysis

**Figure 2:**
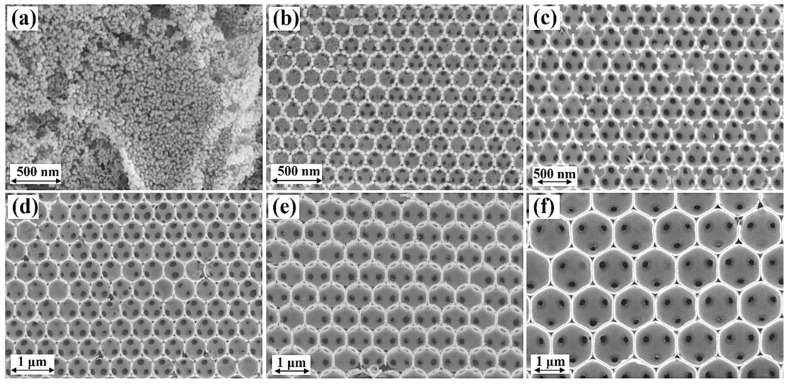
(**a**) FE-SEM image of SnO_2_-TF material, (**b**–**f**) SEM images of SnO_2_-X1 materials (with 0.4 mol/L SnCl_4_·5H_2_O) obtained from different PS template sizes of 200 nm, 500 nm, 700 nm, 1000 nm, and 1500 nm, respectively.

FE-SEM images of PS templates with different sizes are presented in the Supporting Information (Figure S1). These FE-SEM images clearly demonstrate that the spheres are arranged in a highly ordered close-packed structure through a self-assembly process [21]. The uniformity and consistency of this arrangement make it ideal for forming structured templates with well-defined pore sizes. While there are small irregular gaps, it is probable that they were caused by minor defects that were introduced during the stacking process, but the overall structure is mostly highly ordered [22]. It is noteworthy that these PS templates demonstrate the structural integrity that is required for engineering ordered mesoporous materials. FE-SEM images of SnO_2_ samples synthesized both without and with PS templates of varying sizes are shown in Figure 2.

**Figure 3:**
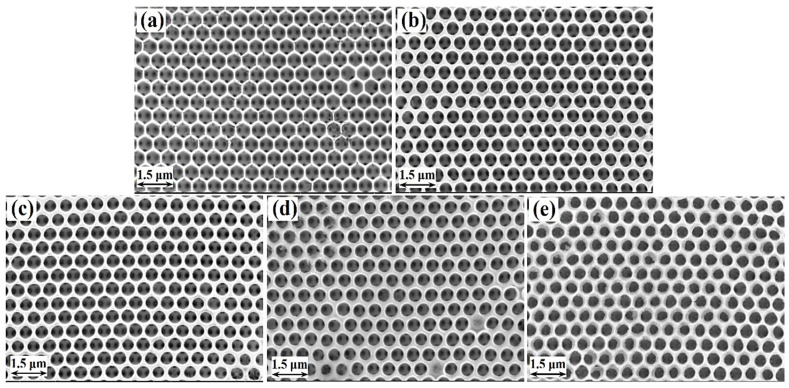
(**a**–**e**) SEM images of SnO_2_-X2 materials (with a 1000 nm PS template size) obtained from different precursor solution concentrations of 0.2, 0.3, 0.4, 0.5, and 0.6 mol/L, respectively.

As shown in Figure 2a, the template-free SnO_2_ (SnO_2_-TF) consists of abundant loosely packed fine particles, forming an irregular mesoporous structure because of uneven aggregation of the particles. In contrast, Figure 2b–f clearly demonstrate the highly ordered structures of SnO_2_-X1 materials synthesized using PS templates. In particular, all the samples exhibit a well-defined and densely packed structures composed of hollow ring-like SnO_2_ frameworks, formed through the removal of 3D PS templates during high-temperature annealing, with a subsequent layer visible underneath the top layer featuring smaller pores. In particular, the removal of PS templates results in the formation of ordered interconnected ring-like SnO_2_ structures, with the size of the resulting pores increasing proportionally to the diameter of the PS templates used. The ring-like appearance in FE-SEM images reflects the top-down view of the interconnected inverse opal voids. It is noteworthy that the diameters of the resulting pores remaining after the annealing process are slightly smaller than those of the original spheres (Figure S1), which is mainly attributed to the structural shrinkage during the transformation of the precursor into SnO_2_ [23]. Additionally, the presence of smaller pores between the ring-like structures and surface irregularities within the SnO_2_ walls contributes to a mesopore structure, which improves the surface area (as discussed later in the BET study) and facilitates effective pathways for gas diffusion. On the other hand, FE-SEM images of SnO_2_-X2 materials synthesized using different precursor concentrations (0.2 to 0.6 mol/L, Figure 3) demonstrate a well-ordered porous network that is different from the one depicted in Figure 2. In particular, after annealing, the removal of 3D PS templates results in the formation of interconnected net-like SnO_2_ structures that resemble nanonets, with a subsequent layer visible underneath the top surface. However, it is noteworthy that the thickness of the nanonet walls increases gradually with higher precursor concentration, resulting in thicker and more robust net walls of SnO_2_ and a corresponding reduction in the diameter of the pores. Herein, an increased precursor concentration leads to an improvement in the mechanical stability and structural robustness of the porous framework through progressive thickening of the SnO_2_ nanonet walls. Moreover, the thickening leads to an increase in the material content, which in turn enhances the density of active sites and enhances gas adsorption capability. While the reduced pore diameter may slightly hamper gas diffusion, the overall ordered porous structure with its interconnected channels and surface area could still promote efficient gas transport. Overall, the balance between mechanical integrity and diffusion efficiency is reflected in this structural evolution, which is crucial for optimizing the material’s performance in applications like gas sensing.

**Figure 4:**
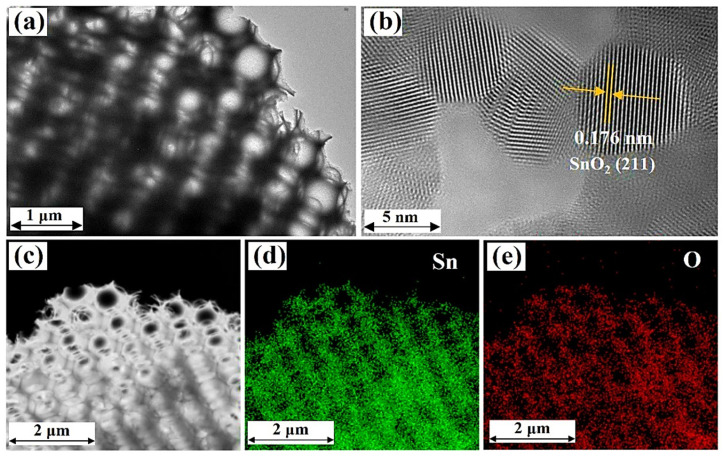
(**a**) Low- and (**b**) high-resolution TEM and (**c**–**e**) TEM mapping images of the SnO_2_-0.4 sample (with a 1000 nm PS template size).

To examine the surface elemental composition, EDX analysis was conducted, and the results are presented in the Supporting Information (Figure S2). Since the SnO_2_-0.4 sample showed the best gas sensing performance (discussed later in sensing section), it was selected for further detailed characterizations like HR-TEM, BET, and XPS. The elemental mapping revealed that prominent peaks belong to tin (Sn) and oxygen (O), demonstrating the successful synthesis of phase-pure SnO_2_ and complete removal of the PS template at the annealing temperature of 550 °C. Notably, the atomic ratio of O to Sn is found to be ~2.65:1, which is higher than the theoretical stoichiometric ratio for SnO_2_ (2:1). This excess oxygen can be credited to the material’s surface area and porous structure, which facilitate higher adsorption of oxygen species on the surface [24]. These adsorbed oxygen species are believed to play a key role in enhancing the sensing performance of the material [12,18,24]. Further insights into the morphology and crystallographic characteristics of the SnO_2_-0.4 sample were gained through TEM analysis, and the corresponding results are presented in Figure 4. In the low-resolution TEM image (Figure 4a), the SnO_2_-0.4 sample clearly exhibits a 3D ordered architecture with a net-like porous profile in the side view, consistent with FE-SEM observations. In particular, TEM reveals moderately exposed spherical voids, overlapping features, and contrast distinctions, confirming a porous network consistent with an inverse opal structure. Notably, these pores (~600 nm in diameter) result from the removal of the 1000 nm PS template, reflecting a ~40% shrinkage in size. Furthermore, the structure clearly shows many mesopores, which create a hierarchical pore structure that is likely to enhance both surface area and gas diffusion efficiency. In contrast, high-resolution TEM (Figure 4b) reveals a well-ordered lattice fringe pattern with a spacing of 0.176 nm, corresponding to the (211) plane of SnO_2_, which is consistent with XRD results. Additionally, TEM mapping images (Figure 4c–e) show a uniform distribution of Sn and O, aligning with the EDS analysis and confirming high elemental homogeneity of the material. This structural uniformity could play a crucial role in its gas sensing performance.

**Figure 5:**
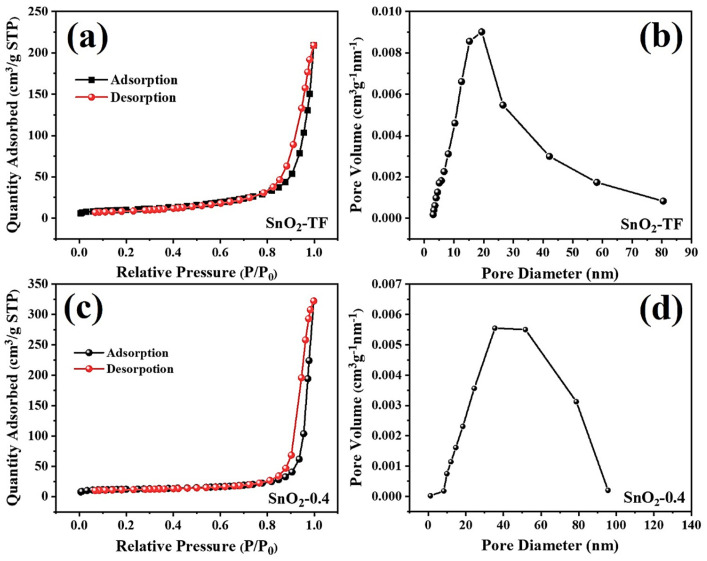
(**a**) N_2_ adsorption/desorption isotherm and (**b**) BJH pore size distribution plot of the SnO_2_-TF sample. (**c**) N_2_ adsorption/desorption isotherm and (**d**) BJH pore size distribution plot of the optimized SnO_2_-0.4 sample (with a 1000 nm PS template size).

To examine the impact of the templating process on the material’s textural properties, the surface area of the optimized SnO_2_-0.4 sample was compared with that of the template-less SnO_2_-TF sample. The BET specific surface area, calculated using N_2_ adsorption/desorption isotherms, of the SnO_2_-0.4 sample (~47 m^2^/g, Figure 5a) is higher than that of the SnO_2_-TF (~36 m^2^/g, Figure 5c) sample, demonstrating the positive effect of the templating process on the development of the ordered porous structure. Moreover, the average pore size calculated by the BJH distribution plot of the SnO_2_-TF (Figure 5b) material increased from 21.91 nm in the template-less sample to ~28 nm in the templated SnO_2_-0.4 (Figure 5d) material, indicating the formation of a more open and accessible mesoporous network. This increase in pore size is a sign that the templating process has resulted in the formation of a more open and accessible mesoporous network. Overall, the texture properties of SnO_2_ samples are improved by employing a PS template during synthesis, resulting in a higher specific surface area and larger pore size. These enhancements are expected to enhance gas sensing performance by providing more active sites and facilitating faster gas diffusion.

### 3.3. XPS Analysis

**Figure 6:**
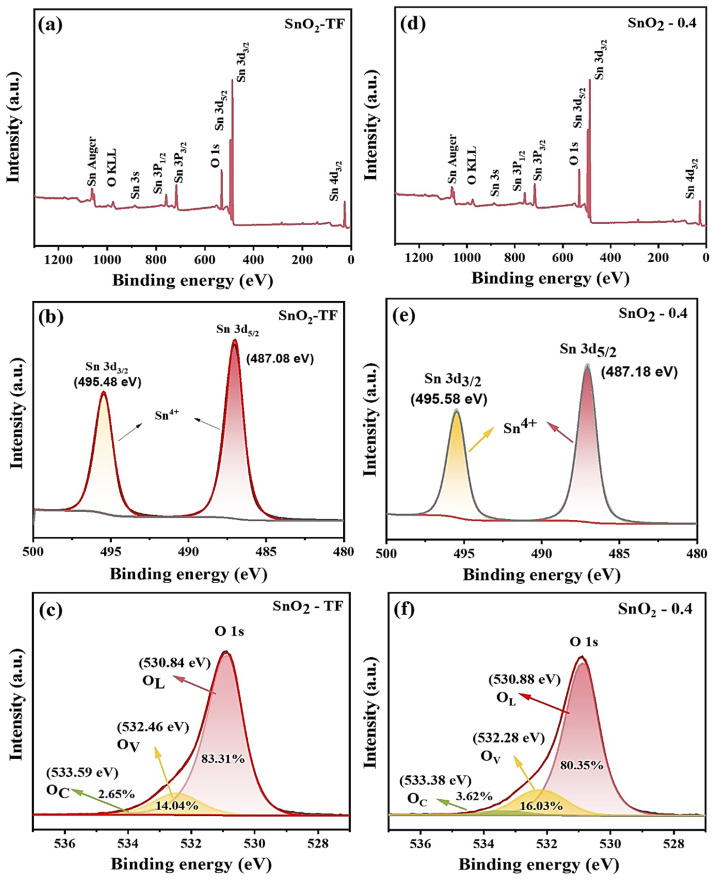
(**a**,**d**) Survey spectrums, (**b**,**e**) Sn 3d core energy level spectrums, and (**c**,**f**) O 1s core energy level spectrums of the SnO_2_-TF and SnO_2_-0.4 samples (with a 1000 nm PS template size), respectively.

To examine the chemical composition and surface states, XPS studies were performed on the optimized SnO_2_ samples prepared with (SnO_2_-0.4) and without (SnO_2_-TF) the PS template, and the results are presented in Figure 6. Notably, the similarities in the XPS patterns of both samples indicate that the chemical composition and oxidation states of both samples are consistent. However, the surface chemistry is characterized by small differences, particularly in the oxygen-related species. A wide energy survey spectrum of both SnO_2_-TF (Figure 6a) and SnO_2_-0.4 (Figure 6d) samples confirm that the presence of peaks belongs only to Sn and O elements, confirming successful removal of the PS template and the formation of pure-phase SnO_2_ without contamination. Core-level Sn 3d spectra of SnO_2_-0.4 (Figure 6e) exhibit two prominent peaks at 487.18 eV and 495.58 eV, corresponding to Sn 3d_5/2_ and Sn 3d_3/2_, respectively. The observed spin-orbit splitting of 8.4 eV between Sn 3d_5/2_ and Sn 3d_3/2_ is characteristic of the +4 oxidation state of Sn [25,26]. Additionally, a core-level O 1s spectrum of SnO_2_-0.4 (Figure 6f) shows three deconvoluted peaks, such as lattice oxygen (O_L_) at 530.88 eV, oxygen vacancies (O_V_) at 532.28 eV, and adsorbed oxygen species (O_C_) at 533.38 eV, with relative areas of 80.35%, 16.03%, and 3.62%, respectively [25,26,27,28]. Herein, the high concentration of lattice oxygen clearly indicates a high degree of crystallinity of SnO_2_. Notably, compared with the core-level O 1s spectrum of template-free SnO_2_-TF sample, the SnO_2_-0.4 sample shows increased concentrations of O_V_ (from 14.04% to 16.03%) and O_C_ (from 2.65% to 3.62%). Thus, despite having similar chemical composition, the SnO_2_-0.4 sample is expected to exhibit better sensing performance due to a higher concentration of surface defect sites and adsorbed oxygen species, which are known to improve sensor response and promote more active surface reactions [28].

### 3.4. Gas Sensing Properties

**Figure 7:**
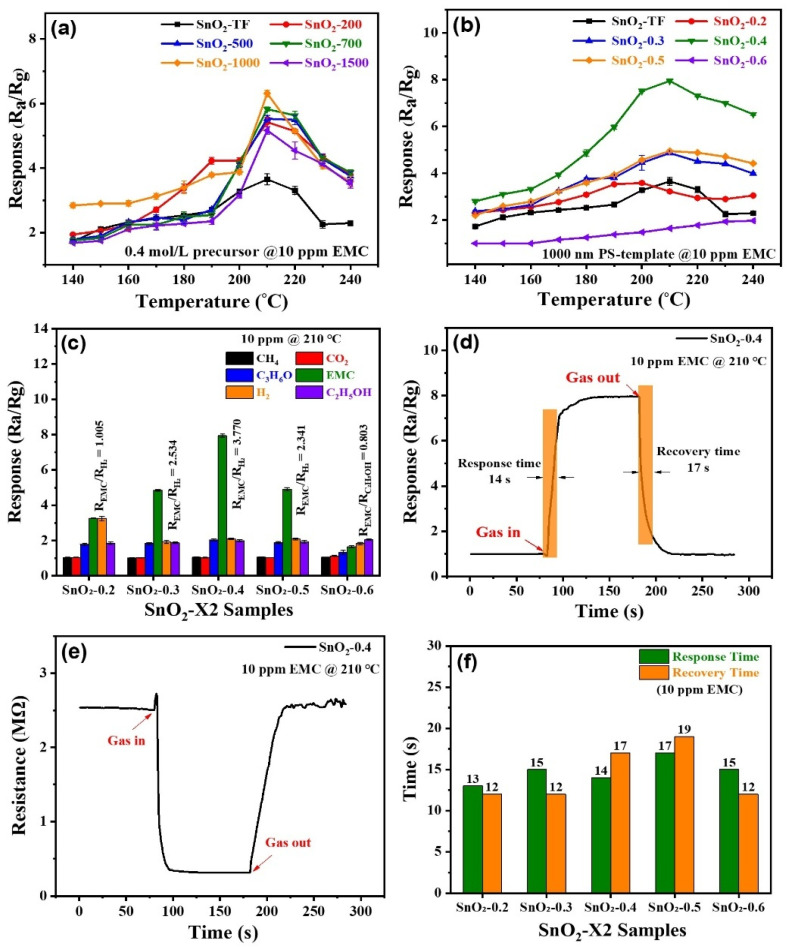
(**a**,**b**) Temperature-dependent response plots of SnO_2_-TF, SnO_2_-X1 (with 0.4 mol/L SnCl_4_·5H_2_O), and SnO_2_-X2 sensors (with a 1000 nm PS template size). (**c**) Selectivity histogram of SnO_2_-X2 sensors, (**d**,**e**) response–recovery plots of the SnO_2_-0.4 sensor, and (**f**) response–recovery time histogram of SnO_2_-X2 sensors.

To evaluate the gas sensing capabilities of the synthesized 3D ordered porous SnO_2_ nanostructures, we systematically investigated how PS template size and precursor concentration affected their performance. Given the mesoporous structure of SnO_2_, we aimed to investigate how precursor concentration, morphology, and synthesis conditions influence its gas sensing characteristics. This approach is crucial for optimizing the structural and surface properties of SnO_2_, thereby enhancing its sensitivity and selectivity toward target gases in gas sensing applications [28,29,30]. Initially, the sensing properties of as-fabricated SnO_2_-X1 (with PS templets) and SnO_2_-X2 sensors (with different precursor concentrations) were studied towards the targeted EMC gas to determine their optimum operating temperatures. In particular, the sensing tests were conducted across a temperature range of 140 °C to 240 °C at a fixed EMC concentration of 10 ppm, and the equivalent results are presented in Figure 7a,b. As shown in Figure 7a, all the SnO_2_-X1 sensors started responding to EMC at 140 °C, reached their maximum response at 210 °C, and then showed a decay in response to a further increase in the temperature. This behavior is basically credited to the desorption of EMC molecules from the sensor surface at higher temperatures, which accordingly reduces the effective gas–sensor interaction [29]. Among various SnO_2_-X1 sensors, the SnO_2_-1000 sensor showed the highest response value of about 6.3, demonstrating that a PS template size of 1000 nm yields the optimal gas sensing performance. Therefore, 1000 nm was selected as the optimal PS template size, and subsequent SnO_2_-X2 materials were synthesized using this template to further investigate the effect of precursor concentration on their gas sensing properties. The temperature-dependent gas sensing performance of SnO_2_-X1 sensors clearly shows that the template size has influenced the sensor performance. As shown in Figure 7a, the EMC sensing response of the SnO_2_-X2 sensors increases with the increase of PS template sizes from 200 nm to 1000 nm. This improvement is mostly attributed to the corresponding increase in the size of the resulting pores, which improves gas diffusion pathways and facilitates more efficient transport of EMC molecules to the active sites on the material surface. However, the EMC sensing response decreases as the PS template size increases from 1000 nm to 1500 nm. This decay is probably due to the excessive enlargement of the pore structure, which reduces the thickness of the internal pore walls of the nanonets. Subsequently, the number of active sites decreases, which leads to weakened gas adsorption and a lower sensing performance. Notably, as shown in Figure 7b, the optimum operating temperatures of the SnO_2_-X2 sensors vary slightly with precursor concentration. Specifically, the SnO_2_-0.2 sensor exhibits its highest gas response at 200 °C and SnO_2_-0.6 at 240 °C, while the other sensors (SnO_2_-TF, SnO_2_-0.3, SnO_2_-4, and SnO_2_-5) demonstrate optimal performance at 210 °C. Overall, among all the SnO_2_-X2 sensors, the SnO_2_-0.4 sensor exhibits the highest response value of 7.95 to 10 ppm EMC, followed by the SnO_2_-0.5 and SnO_2_-0.3 sensors. In contrast, the SnO_2_-0.2 and SnO_2_-0.6 sensors show lower response values than the SnO_2_-TF sensor. The structural formation and gas sensing properties of SnO_2_ frameworks are influenced by precursor concentration, and these variations can be attributed to this influence. For instance, at moderate precursor concentrations (0.3–0.5 mol/L), the precursor solution can fully infiltrate the PS templates, forming an 3D ordered mesoporous structure. This optimal architecture boosts the number of surface oxygen vacancies and active sites, thereby improving gas adsorption and enabling the surface reactions critical to sensing performance. However, a low precursor concentration (0.2 mol/L) results in inadequate infiltration and suboptimal porous networks, leading to a reduced number of active sites and diminished sensing ability. Conversely, an excessively high concentration (0.6 mol/L) produces overly thick pore walls, which hinder gas diffusion paths and therefore lower the gas response. These results highlight the importance of fine-tuning precursor concentration to balance structural integrity and surface reactivity for optimized gas sensing performance. Overall, a comparison of the temperature-dependent gas sensing responses of SnO_2_-X1 and SnO_2_-X2 sensors reveal that the SnO_2_-0.4 sensor shows the highest EMC response at 210 °C, which is selected as the optimal sensing temperature for the following studies. Afterwards, a selectivity study was conducted on the SnO_2_-X2 sensors towards a fixed 10 ppm concentration of various target gases at an optimal working temperature of 210 °C, and the corresponding results are presented in Figure 7c. The selected gases for selectivity study include methane (CH_4_), hydrogen (H_2_), acetone (C_3_H_6_O), ethanol (C_2_H_5_OH), carbon dioxide (CO_2_), and EMC, which are representative of key electrolyte leakage gases generated during TR events in LIBs [4,8]. Among various SnO_2_-X2 sensors, all the sensors, except SnO_2_-0.2 and SnO_2_-0.6, showed good selectivity toward EMC (Figure 7c). The reduced selectivity observed in the SnO_2_-0.2 and SnO_2_-0.6 sensors may be attributed to the suboptimal precursor concentration. The microstructure of the sensing material can be negatively affected by either too low or too high of a precursor concentration, resulting in an imbalance in surface chemistry and a decrease in response and selectivity towards the target gas. Notably, the SnO_2_-0.4 sensor demonstrated the highest selectivity for EMC at 210 °C as its response to 10 ppm EMC was significantly higher than other gases. In particular, the response to 10 ppm EMC was 3.77 times greater than its response to 10 ppm H_2_ (Figure 7c). This indicates excellent selectivity and strong resistance to interference from other gases, highlighting the good sensing performance of the SnO_2_-0.4 sensor towards EMC gas. While the sensor demonstrates promising EMC sensing performance, it operates at a high temperature of 210 °C, a known limitation of SMOs-based sensors, which we aim to overcome in future studies by decorating SnO_2_ surface with noble metal catalysts to enable low temperature operation [8,16].

**Figure 8:**
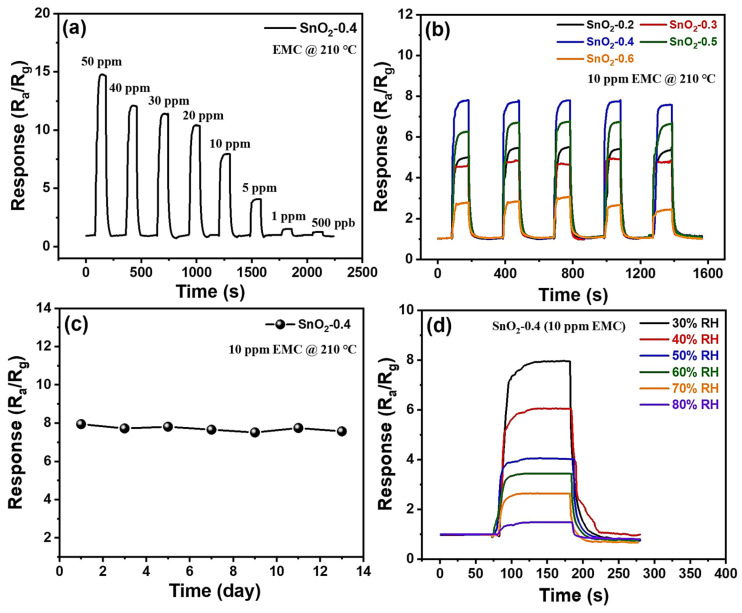
(**a**) A transient response plot of the SnO_2_-0.4 sensor (with a 1000 nm PS template size) towards different EMC levels, (**b**) reproducibility study of the SnO_2_-X2 sensors (with a 1000 nm PS sphere size) towards 10 ppm EMC, (**c**) response stability test of the SnO_2_-0.4 sensor, and (**d**) dynamic response curves of the SnO_2_-0.4 sensor under different humidity levels.

Figure 7d,e displays the response and corresponding resistance–time plots of the optimized SnO_2_-0.4 sensor upon exposure to 10 ppm EMC gas at 210 °C, respectively. These plots were used to determine the response and recovery times of the sensor. As shown in Figure 7d, when exposed to 10 ppm of EMC, the SnO_2_-0.4 sensor exhibits a rapid increase in the response value from approximately 1.0 to a peak of ~7.95. Correspondingly, the resistance value of the sensor decreases from an initial value of ~2.52 MΩ to a minimum of ~0.31 MΩ (Figure 7e) with rapid response and recovery times of 14 s and 17 s, respectively. The response and recovery times of all SnO_2_-X2 sensors have been compared, and the results are shown in Figure 7f. These results demonstrate that the response times range from 13 to 17 s, while recovery times fall within 12 to 19 s. The observed results clearly indicate that the SnO_2_-X2 sensors, particularly the SnO_2_-0.4 sensor, have both rapid response/recovery features, which are crucial for the timely detection of EMC emissions linked with early thermal runaway in LIBs. Subsequently, the effect of varying EMC concentrations, ranging from 50 ppm to 500 ppb, on the optimized SnO_2_-0.4 sensor was studied, and the equivalent response transient curves are presented in Figure 8a. As shown in Figure 8a, the sensor’s response value decreases gradually with decreasing EMC levels from 50 ppm to 500 ppb at 210 °C. Such a decrease in sensor response at lower concentrations can typically be attributed to the reduced availability of gaseous molecules, which limits the interaction between the gas and the sensor surface, thereby diminishing the sensor’s response. Remarkably, the SnO_2_-0.4 sensor exhibits a distinct and measurable response of ~1.26, even at a low concentration of 500 ppb. However, at EMC levels below 500 ppb, the sensor response becomes very weak and nearly indistinguishable, demonstrating that the detection limit of the SnO_2_-0.4 sensor is 500 ppb. Thus, the SnO_2_-0.4 sensor, with its low detection capability of 500 ppb, is suitable for detecting low levels of EMC, making it a promising candidate for sensor development in LIB safety applications. To evaluate the repeatability and stability of the SnO_2_-X2 sensors, the sensing tests were conducted at an optimized temperature of 210 °C with a fixed 10 ppm concentration of EMC. Figure 8b shows that all SnO_2_-X2 sensors demonstrated excellent repeatability over five successive EMC exposure cycles, with each cycle exhibiting complete recovery features. These results confirm that the SnO_2_-X2 sensors can sustain consistent performance efficiently, even after repeated exposure to EMC gas. The stability study of the optimized SnO_2_-0.4 sensor is shown in Figure 8c, where the sensor was subjected to 10 ppm EMC gas every 24 h over a period of 13 days under the same operating conditions. The stability analysis revealed that the sensor response remained stable throughout the tests, with minor fluctuations below 4%, indicating that performance did not decline over time.

To evaluate the response stability of the sensor under realistic humidity conditions, the sensing performance of the optimized SnO_2_-0.4 sensor was studied at 210 °C in a 10 ppm EMC atmosphere, while varying the relative humidity (RH) from 20% to 80% using a humidifier and dehumidifier. As shown in Figure 8d, the SnO_2_-0.4 sensor exhibited its highest response value of 7.95 at 30% RH. However, as the humidity level increased, the sensor response declined significantly, reaching a value of ~1.5 at 80% RH. The sensor performance is significantly impacted by changes in humidity, primarily due to its high surface area and 3D network of ordered mesoporous nanonets, which enhance its sensitivity to moisture. In particular, water molecules can compete for adsorption with the target EMC molecules by adsorbing and condensing on both the material surface and within its pores. This competition has an impact on the gas sensing reaction, potentially affecting the conductivity of the sensing material and certainly blocking gas transport channels [30,31,32]. These effects collectively contribute to the reduced response and inadequate humidity stability of the SnO_2_-0.4 sensor under high humidity conditions. Our future efforts will therefore be directed towards improving the reliability of our sensor in humid conditions by reducing the effect of water vapor by decorating the sensor surface using noble metal nanoparticles. These noble metals are expected to reduce the interference caused by water vapor by enhancing the sensor’s selectivity and promoting more stable interactions with EMC molecules [33]. Moreover, alternative strategies like hydrophobic surface treatments or polymer overlayers can also aid in mitigating humidity effects on SMOs-based gas sensors. While these modifications may influence surface interactions, careful material selection and optimized synthesis techniques can preserve gas sensitivity and selectivity of the sensor. Moreover, incorporating a water-selective filtration membrane above the sensor offers a promising approach to effectively reduce humidity interference.

Finally, we have compared the sensing performance of our optimized SnO_2_-0.4 nanonet sensor with previously reported EMC sensors (see Table 1) [6,13,14]. As shown in Table 1, the work by Su et al. [6] reports better performance in terms of sensitivity, operating temperature, and detection limit for EMC sensing. However, our developed SnO_2_-0.4 nanonets sensor offers a distinct advantage in terms of significantly faster response and recovery times, which is a critical parameter for real-time gas detection applications. Such sensor dynamics is particularly valuable for rapidly detecting electrolyte-based carbonate gases emitted during the early stages of TR events in batteries, enabling timely intervention and enhancing battery safety. Overall, these results highlight the effectiveness of the nanonet architecture in enhancing gas sensing performance, making the SnO_2_-0.4 sensor a solid candidate for the development of sensors for EMC detection in battery safety applications. Nonetheless, our future studies will focus on further improving sensitivity, lowering the detection limit and operating temperature of the sensor while maintaining the fast response and recovery characteristics through the above-mentioned strategies. This integrated approach aims to develop a more efficient and high-performance EMC sensing platform.

### 3.5. Gas Sensing Mechanism

**Scheme 2:**
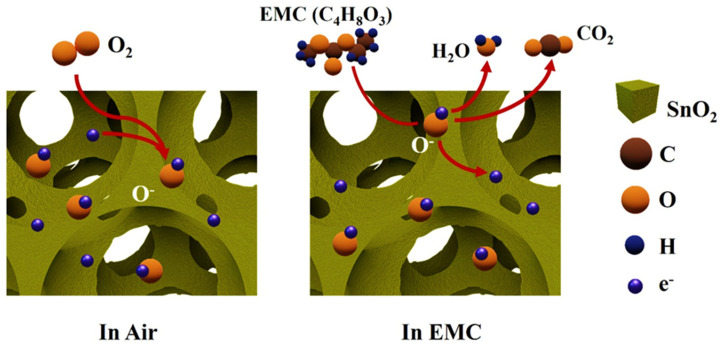
Schematic representation of sensing interactions between SnO_2_ and EMC molecules.

It is well known that the sensing mechanism of chemiresistive gas sensors based on SMOs is primarily dependent on the adsorption and desorption of target gas molecules on the sensor surface, which modulates its electrical resistance [34,35]. In particular, during this process, oxygen plays a crucial role by forming surface adsorbed oxygen species that interact with target gases, leading to a change in charge carrier concentration and thus changing the resistance of the sensor material [34,35,36,37]. In this study, from gas sensing tests, we observed that the SnO_2_ sensors showed a significant decrease in resistance when exposed to reducing EMC molecules (Figure 7e), which is the characteristic of n-type semiconductors [6]. This phenomenon is clarified in the following way. When an ordered porous SnO_2_ sensor is exposed to air, oxygen molecules are adsorbed onto the surface of the sensor by capturing electrons from the conduction band of SnO_2_, leading to the formation of temperature-dependent negatively charged oxygen species, such as O_2_^−^, O^−^, or O^2−^. Herein, at 210 °C, O^−^ species are the dominant adsorbed oxygen species (see Scheme 2). Due to the electron withdrawal, an electron depletion layer is generally formed near the surface, which typically increases the sensor’s resistance. When exposed to EMC, this reducing gas interacts with formerly adsorbed oxygen species on the inner and outer surfaces of the material, leading to the formation of H_2_O and CO_2_. This reaction releases trapped electrons back into the conduction band of SnO_2_, thereby increasing the carrier concentration and resulting in a measurable drop in the electrical resistance of the sensor. This reversible interaction supports the gas sensing response of SMOs-based sensors. The possible interactions between adsorbed oxygen and EMC molecules are as follows [6]:

(1) $$O_{2(gas)}+{2e}^{-}↔{2O}_{\left(ads\right)}^{-}$$

(2) $$C_{4}H_{8}O_{3(gas)}+{9O}_{\left(ads\right)}^{-}↔{4CO}_{2}+{4H}_{2}O+9e^{-}$$

Although the sensing mechanism discussed above provides a foundation, the sensing process could be more complex and extend beyond the simplified reaction equations. On the SnO_2_ surface, multiple dynamic processes may occur simultaneously, including the adsorption/desorption of oxygen species, EMC molecules, and water vapor, as well as potential secondary reactions such as partial adsorption or decomposition of EMC. Future studies, possibly including in situ spectroscopic characterization techniques, could help to gain deeper insights into these mechanisms. In summary, the observed sensing performance of the SnO_2_-0.4 sensor is mainly attributed to an ordered mesoporous nanonet-type structure and specific surface area (~47 m^2^/g), which together facilitated the efficient diffusion of EMC gas molecules to both the surface and internal regions through an increased surface-to-volume ratio and well-defined transport channels that optimize diffusion pathways. As a result, the contact efficiency between the EMC gas molecules and the material surface has been significantly enhanced. Furthermore, the abundant mesopores advanced the materials gas adsorption capacity by increasing the exposure of active sites. Moreover, the ordered pore network of nanonets also facilitated the formation of surface oxygen vacancies and chemisorbed oxygen species, which acted as active sites for EMCs adsorption, thereby allowing stronger interaction with the gas molecules and enhancing gas response towards EMC [6,38,39]. Overall, these findings underscore the importance of structural and compositional control in the development of gas sensors for the early detection of EMC released during TR in LIBs, providing a valuable strategy for enhancing battery safety.

## 4. Conclusions

In conclusion, 3D ordered porous SnO_2_ nanostructures were successfully synthesized via a simple PS templating method by systematically varying both the template sizes (SnO_2_-X1 series) and precursor concentrations (SnO_2_-X2 series), allowing precise control over pore structure and surface properties to enhance EMC sensing performance. Initially, PS-templated SnO_2_-X1 samples, prepared using PS diameters ranging from 200 to 1500 nm, were employed to tailor the pore architecture of the SnO_2_ materials. Among them, the SnO_2_-1000 sample—synthesized using 1000 nm PS size and a 0.4 mol/L precursor solution—exhibited the most definite pore connectivity and optimized surface characteristics, with the ordered nanostructure resembling ring-like surface morphologies. This SnO_2_-1000 sample exhibited a good gas response of 6.3 (10 ppm EMC at 210 °C) compared with other template-based and template-free SnO_2_ materials, confirming 1000 nm as the optimal template size. Subsequently, a series of SnO_2_-X2 materials (SnO_2_-0.2 to SnO_2_-0.6) were synthesized using the 1000 nm template and varying precursor concentrations of 0.2–0.6 mol/L. Among them, the nanonet-like SnO_2_-0.4 sample demonstrated an optimal combination of structural integrity and functional performance. It exhibited a well-ordered pore structure with uniform walls, a high surface area (~47 m^2^/g), increased oxygen vacancy and chemisorbed oxygen species, and a mesoporous size distribution centered at ~28 nm. These features correlated directly with improved gas sensing characteristics: a maximum response of ~7.95 to 10 ppm EMC at 210 °C, fast response/recovery times (14/17 s), low detection limit (500 ppb), and excellent response reproducibility and stability. However, the SnO_2_-0.4 sensor experienced a decrease in response under high humidity conditions, which was caused by water molecules adsorption within the porous network, which accordingly disrupts the interaction of EMCs with the sensor. Given the humidity sensitivity of the mesoporous SnO_2_-0.4 sensor, our future efforts will focus on surface engineering to overcome this limitation while sustaining the advantages of the 3D ordered architecture. Overall, this work advances the understanding of EMC sensing using ordered porous SnO_2_ nanostructures and highlights the importance of structural/compositional control in developing gas sensors for the early detection of EMC released during TR in LIBs, offering a valued strategy for enhancing battery safety and a solid foundation for future research in this field.

## Figures and Tables

**Table 1:** Comparison of the EMC sensing performance of the SnO_2_-0.4 sensor with previously reported sensors.

Materials	Target Gas	Gas Concentration (ppm)	Response	Response/ Recovery Times (s)	Optimal Operating Tempt. (°C)	Lowest Detection Limit (ppb)	Ref.
SnO_2_ nanobox	EMC	10	19	71/257	140	10	[6]
SnO_2_ nanocage	EMC	10	6.92	100/983	140	160	[13]
WO_3_ hollow microsphere	EMC	10	5.3	45/799	275	100	[14]
SnO_2_-0.4 nanonets	EMC	10	7.95	14/17	210	500	This work

## Data Availability

The data that support the findings of this study are available from the corresponding authors upon reasonable request.

## Supplementary Materials

The following supporting information can be downloaded at: https://www.mdpi.com/article/10.3390/nano15151150/s1, Figure S1: FE-SEM images of PS templates with different sizes: (a) 200 nm, (b) 500 nm, (c) 700 nm, (d) 1000 nm, and (e) 1500 nm; Figure S2: (a–d) Elemental mapping images and (e) EDX spectrum of the SnO_2_-0.4 sample.
