# Supplementary material for: Three-Dimensional Ordered Porous SnO2 Nanostructures Derived from Polystyrene Sphere Templates for Ethyl Methyl Carbonate Detection in Battery Safety Applications
Source: Nanomaterials (Basel). 2025 Jul 25;15(15):1150. doi: 10.3390/nano15151150 (PMC12348349; doi:10.3390/nano15151150)
Supplement: Supplementary file 1 [file nanomaterials-15-01150-s001.zip › nanomaterials-3767026-supplementary.pdf]

Supporting Information

Three-Dimensional Ordered Porous SnO_2 Nanostructures Derived from Polystyrene Sphere Templates for Ethyl Methyl Carbonate Detection in Battery Safety Applications

---

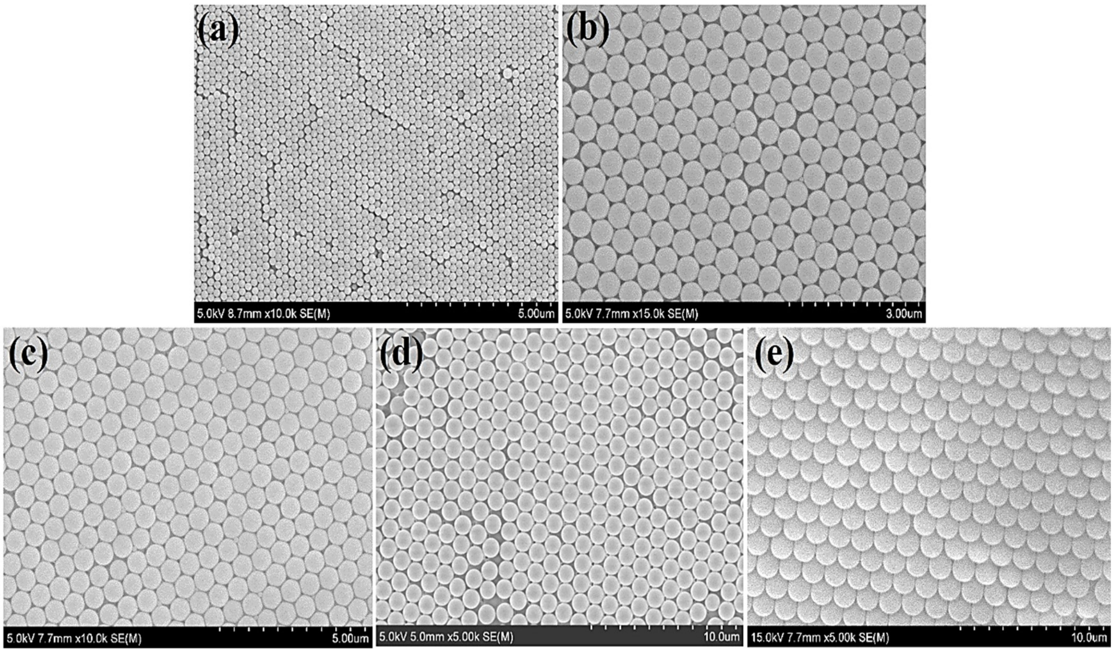

Figure S1: FE-SEM images of PS-sphere templates with different sizes (a) 200 nm, (b) 500 nm, (c) 700 nm, (d) 1000 nm, and (e) 1500 nm.

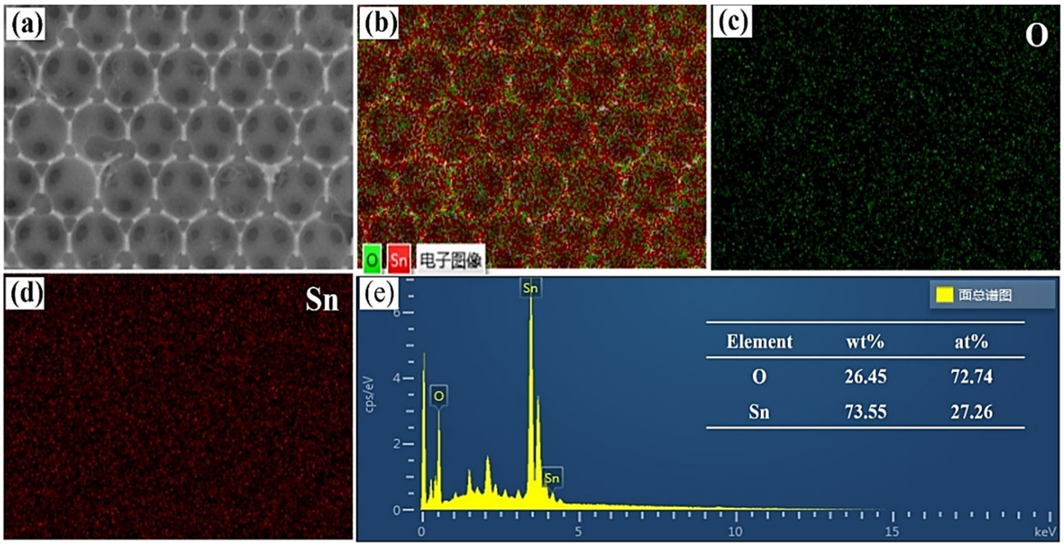

Figure S2: (a-d) Elemental mapping images and (e) EDX spectrum of SnO_2-0.4 sample (with 1000 nm PS-sphere size).
